# Systemic lupus erythematosus with recurrent posterior reversible encephalopathy syndrome: A rare case report

**DOI:** 10.1097/MD.0000000000043428

**Published:** 2025-07-18

**Authors:** Binbin Chen, Hong Hong, Jie Wang, Xiuzhen Li, Cong Wang, Tingting Xie

**Affiliations:** a Department of Nephrology, Liaocheng People’s Hospital, Liaocheng, Shandong Province, China; b Department of Pediatric Nephrology, Shandong Provincial Hospital, Jinan, Shandong Province, China.

**Keywords:** lupus nephritis, posterior reversible encephalopathy syndrome, systemic lupus erythematosus

## Abstract

**Rationale:**

Posterior reversible encephalopathy syndrome (PRES) is a rare but potentially life-threatening neurological condition. In patients with systemic lupus erythematosus (SLE), PRES can occur as a complication of hypertension, renal dysfunction, or immunosuppressive treatment, and is often difficult to distinguish from neuropsychiatric lupus.

**Patient concerns:**

A 17-year-old woman was diagnosed with SLE, presenting with fever, hypertension, and severe lower limb edema. Laboratory investigations revealed low serum complement levels, positive ANA and ds-DNA, nephrotic range proteinuria, elevated serum urea and creatine, anemia, and thrombocytopenia.

**Diagnoses:**

SLE with PRES.

**Interventions:**

The patient promptly received methylprednisolone pulse therapy and cyclophosphamide for the lupus flare, along with symptomatic and supportive treatment, including antiepileptic drugs, blood pressure control, and renal replacement therapy. Her condition improved with the resolution of imaging features of PRES within 2 weeks. However, 8 weeks later, seizures recurred, and a repeat brain magnetic resonance imaging revealed a worsening of PRES. Notably, laboratory tests did not indicate SLE exacerbation. Blood pressure control was further intensified, and intensive hemodialysis was administered. The induction therapy, including corticosteroids and cyclophosphamide, was continued.

**Outcomes:**

The patient’s PRES symptoms, including headache, seizures, loss of vision, and frequent vomiting, significantly improved. She was successfully discharged and follow-up examinations confirmed the resolution of PRES and stable SLE.

**Lessons:**

The symptoms of PRES in SLE patients can mimic those of neuropsychiatric systemic lupus erythematosus. Timely differential diagnosis and appropriate treatment are crucial to reduce the recurrence of PRES and improve patient prognosis.

## 1. Introduction

Systemic lupus erythematosus (SLE) is the prototypical systemic autoimmune disease that can involve virtually every organ and tissue in the body. Up to 50% of patients with SLE may develop neurological signs or symptoms during their disease course.^[1]^ Neuropsychiatric systemic lupus erythematosus (NPSLE) is a complex and nonspecific manifestation that can occur during both active and resting stages of SLE. It is one of the most potentially disabling and least understood aspects of the disease. Although 19 neuropsychiatric syndromes have been described in SLE, posterior reversible encephalopathy syndrome (PRES) is not typically classified as one of them.^[2]^ PRES is a clinical-radiological entity characterized by symptoms such as headache, confusion, seizures, and visual impairment. Radiologically, PRES is marked by bilateral symmetrical vasogenic edema in the posterior white matter. PRES is associated with several disorders, including hypertension, autoimmune diseases, renal failure, cytotoxic drug therapy, and infections. The physiopathology of PRES is not completely understood. The most accepted hypotheses are based on abnormalities in blood vessel regulation in the central nervous system.^[3]^ Although PRES is generally considered reversible, with prompt diagnosis and treatment often leading to full recovery, the condition can be fatal in some cases.^[4]^ Here we report a case of SLE complicated by recurrent PRES. Our experience with this patient may provide insights for managing SLE with PRES in clinical practice.

## 2. Case presentation

A 17-year-old woman was admitted to our hospital on 23 December 2022 with intermittent fever and bilateral leg edema for 6 days. Physical examination revealed highest body temperature of 39.0℃, blood pressure of 150/100 mm Hg, oxygen saturation of 98% while breathing ambient air, sporadic moist rales, sinus tachycardia (heart rate 105 bpm), and grade 3 lower-extremity pitting edema. Neurological examination was normal. Meningeal signs were negative. Her serum albumin level was 2.6 g/dL (26 g/L), serum creatinine level was 5.82 mg/dL (515 μmol/L), white blood cell (WBC) count was 6620/μL, neutrophil count was 5290/μL, hemoglobin was 57 g/L, and platelet count was 4400/μL. Urinary protein excretion was 3.6 g/d. Serological tests for antinuclear antibody (ANA; 1:000, speckled pattern), anti-Smith (Sm) antibody, anti-SS-A antibody, and anti-double-stranded DNA (ds-DNA) antibody were positive. The Coombs test was also positive. She had low serum complement levels (C3 0.111 g/L, C4 0.0245 g/L). Anti-neutrophil cytoplasmic antibody, anti-glomerular basement membrane antibody, antiphospholipid antibody, hepatitis B surface antigen, and hepatitis C antibody were negative. Antistreptolysin O titer, and rheumatoid factor were within the normal range. Urine microscopy revealed 50 to 80 RBCs and 3 to 8 WBCs per high-power field. Electrocardiogram showed a small amount of pericardial effusion and an ejection fraction of 65%. Thoracic and abdominal computed tomography findings were suggestive of pneumonia and seroperitoneum; the appearance and size of the kidneys were normal. Doppler ultrasound showed no signs of deep vein thrombosis in bilateral lower extremities.

**Figure 1.**
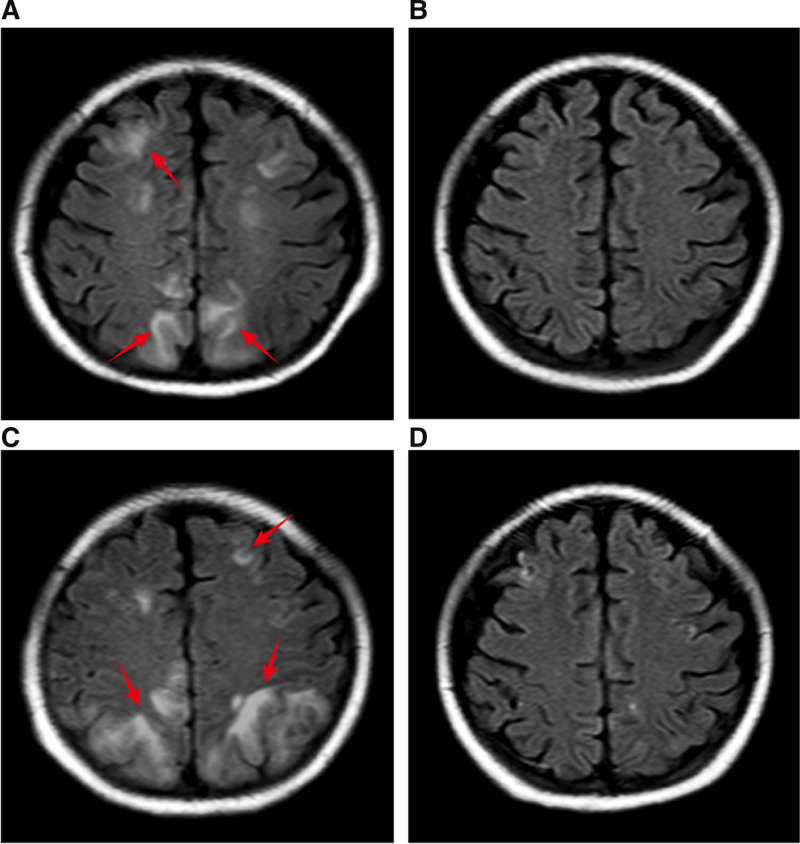
Seventeen-year-old female with 2 episodes of PRES. MRI during the first episode shows hyperintense lesions of the bilateral frontal and parieto-occipital (A). On a control MRI 2 wk later, the lesions have resolved completely (B). After 8 wk without any symptoms, the patient was admitted again because of a generalized seizure. MRI shows hyperintense lesions again that reveals the recurrence of PRES (C). With meticulous blood pressure control, reinforced hemodialysis and ultrafiltration, antiepileptic medication, and therapies to protect brain cells, repeat brain MRI showed significant improvement of PRES (D). MRI = magnetic resonance imaging, PRES = posterior reversible encephalopathy syndrome.

Based on clinical and laboratory findings, a diagnosis of SLE was considered with multi-organ involvement. Renal involvement manifested as lupus nephritis (LN), nephritic syndrome, and acute renal failure; hemopoietic system involvement manifested as hemolytic anemia, thrombocytopenia, and serous membrane effusion. She also had a complication of pneumonia. She was admitted to the kidney intensive care unit for lupus flare and progressive oliguria for the next 2 days, where she received intravenous methylprednisolone 500 mg once daily for 3 days, cyclophosphamide 0.4 g stat and γ-globulin 20 g once daily for 5 days; this was followed by 1 mg/kg oral prednisone daily. In addition, she underwent intermittent hemodialysis and blood transfusion for symptomatic treatment. One week after treatment, the edema was resolved, her body temperature dropped to normal, hemoglobin levels returned to 80 g/L, and the platelet count increased to 7800/μL. However, there was no improvement in renal function and she remained hemodialysis-dependent. Overall, the general status of the patient seemed to improve. However, ten days after admission, the patient experienced 2 episodes of tonic-clonic seizures within a span of 2 hours, manifesting as sudden generalized convulsions, loss of consciousness, upturned eyes, and foaming at the mouth. Intravenous administration of diazepam led to the resolution of symptoms. On brain MRI, T2/fluid-attenuated inversion recovery sequence showed signal hyperintensity in the bilateral frontal and parieto-occipital (Fig. 1A). These clinical and radiological findings confirmed the diagnosis of PRES. Subsequently, she became confused, with waxing and waning consciousness, and developed headaches and frequent retching. Her blood pressure was high during and after the seizure episode (200/110 mm Hg). Antihypertensive treatment was reinforced with continuous intravenous pump infusion of sodium nitroprusside and then transitioned to oral valsartan-amlodipine and carvedilol, with close monitoring of blood pressure below 140/90. Immunosuppressive therapy was continued with prednisone at a dose of 50 mg/d. Her mentation gradually improved over a period of 1 week. Repeat brain MRI after 2 weeks (Fig. 1B) revealed near resolution of lesions. After a hospital stay of 4 weeks, her condition improved and she was discharged from the hospital, with tapering oral prednisone 50 mg/d, cyclophosphamide 0.4 g monthly, hydroxychloroquine 200 mg daily, topiramate 100 mg twice a day, valsartan-amlodipine 80 mg/5 mg daily, intermittent hemodialysis twice a week and control of medication side effects, as needed.

Eight weeks after discharge, on the way to hospital for hemodialysis, the patient developed seizures again. She was taken to the emergency department, where her blood pressure was found to be 230/130 mm Hg. Oral prednisone was reduced to 30 mg/d and the total dose of cyclophosphamide was 1.6 g. The blood pressure control was borderline at home. She was treated with analgesics and anticonvulsants along with strict control of blood pressure below 140/90 mm Hg with intravenous nitroprusside. Repeat brain MRI revealed recurrence of PRES (Fig. 1C), even worse than before. The patient developed aphasia. Physical examination revealed normal body temperature with no rash or purple discoloration. Neurological examination revealed impaired cognitive ability. Vision examination revealed decreased visual acuity in both eyes. Her muscle strength was normal. Kernig’s sign and bilateral Babinski’s sign were negative. Subsequent laboratory examination revealed WBC count within the normal range, hemoglobin of 107 g/L, platelet count of 9000/μL, and an ANA titer of 1:160. Her ds-DNA antibody level declined, while serum complements levels were raised (C3 0.38 g/L, C4 0.12 g/L). Repeat antiphospholipid antibody test and Coombs test were negative. There was no electrolyte disturbance. The patient’s SLE was stable, excluding neurological manifestations, based on the above clinical features and tests. Given the controversy surrounding PRES as a neuropsychiatric manifestation and the absence of evidence indicating active SLE in other organs/systems, the immunosuppressive regimen was not adjusted significantly. Oral prednisone was replaced with intravenous methylprednisolone 40 mg daily and the scheduled cyclophosphamide administration was postponed by 2 weeks due to the patient’s hospitalization for H1N1 influenza and resulting interstitial pneumonia. With meticulous blood pressure control, reinforced hemodialysis and ultrafiltration, antiepileptic medication, and therapies to protect brain cells, the seizures of the patient were controlled along with improvement in aphasia in 4 days. Four weeks after admission, the patient was discharged again, and repeat brain MRI showed significant improvement of PRES (Fig. 1D). As of 8 months post-discharge, her condition has remained stable. Her blood pressure was maintained below 140/90 mm Hg using a combination of valsartan-amlodipine (80 mg/5 mg daily) and carvedilol (25 mg daily). The patient’s SLE remained stable, cyclophosphamide was discontinued for 2 months, and no seizures or lupus flares occurred.

## 3. Discussion

This case describes a 17-year-old female with newly diagnosed SLE who developed PRES in the context of multi-organ involvement, including LN, hemolytic anemia, thrombocytopenia, and acute renal failure. Despite prompt immunosuppressive therapy and supportive management, including methylprednisolone, cyclophosphamide, and renal replacement therapy, the patient experienced seizures and was diagnosed with PRES based on typical clinical presentation and characteristic MRI findings. Although her symptoms improved following intensified antihypertensive treatment and continued immunosuppression, a recurrence of PRES occurred 8 weeks later, despite stable serologic markers of SLE activity. With reinforced blood pressure control, antiepileptic therapy, and supportive care, the patient gradually recovered. This case highlights the diagnostic and therapeutic challenges of managing PRES in patients with active SLE, particularly in distinguishing it from neuropsychiatric lupus and preventing recurrence.

SLE is a highly diverse autoimmune disease capable of affecting any organ. Nervous system involvement is common in SLE patients, but only 13% to 38% of neuropsychiatric events are attributable to the disease itself, known as NPSLE. Other factors, such as infection, drugs, and metabolic imbalances can also lead to neuropsychiatric disorders.^[5]^ While the survival and prognosis of patients with SLE have improved significantly in recent years, NPSLE remains a major cause of morbidity and mortality, second only to LN.^[6]^ It is suggested that SLE patients with both LN and NPSLE may have the poorest prognosis. Because neuropsychiatric manifestations in SLE are nonspecific and heterogeneous, it is crucial to determine whether nervous system involvement is attributable to SLE, non-SLE factors, or both, in order to develop appropriate treatment strategies.

Recent advances in molecular immunology have shed light on the underlying mechanisms of SLE. The mitogen-activated protein kinase signaling pathway has been implicated in the regulation of inflammation and immune responses, both of which are central to SLE pathogenesis. Abnormal activation of mitogen-activated protein kinase cascades contributes to chronic inflammation and immune dysregulation in SLE, and may play a role in the neuroinflammatory processes underlying NPSLE.^[7]^ In addition, mesenchymal stem cells have emerged as a promising approach in the field of regenerative medicine and immunotherapy. Their ability to modulate immune responses, suppress pro-inflammatory cytokines, and enhance regulatory T-cell activity has shown potential in restoring immune homeostasis in autoimmune diseases, including SLE. Recent studies suggest that mesenchymal stem cells may help ameliorate both systemic and central nervous system inflammation in SLE patients.^[8]^

PRES is an uncommon clinicoradiological syndrome characterized by acute cerebral endotheliopathy, leading to the disruption of the blood-brain barrier and subsequent vasogenic edema. First described by Dr Hinchey and colleagues in the New England Journal of Medicine in 1996, PRES has become increasingly recognized due to advancements in imaging modalities, particularly brain MRI.^[3,9]^ PRES typically presents with a range of acute neurological symptoms, including seizures, encephalopathy, headache, and visual disturbances. These clinical manifestations often correlate with characteristic findings on MRI, such as reversible T2 or fluid-attenuated inversion recovery imaging hyperintensities located in the occipital and parietal regions.^[10,11]^ Over the last 2 decades, various diseases and factors have been associated with PRES, such as hypertension, autoimmune disease (especially SLE), renal dysfunction/failure, preeclampsia/eclampsia, posttransplantation status, immunosuppression (e.g., due to corticosteroids, tacrolimus, mycophenolate mofetil), chemotherapy (e.g., with cyclophosphamide, platinum), infection/sepsis, and various other potential causes.^[12,13]^ With early and appropriate treatment, primarily addressing the underlying cause, PRES has a favorable prognosis. However, neurological deficits and even death can occur, particularly in patients who develop less common cytotoxic edema or complications such as intracranial hemorrhage. Recurrence rates of PRES have been reported between 5% and 14%, with poor blood pressure control being a primary contributing factor.^[14,15]^

This SLE patient experienced recurrent PRES within a short time frame. The first episode was attributed to the autoimmune disease and coincided with SLE remission. However, the subsequent PRES episodes were likely multifactorial, involving hypertension, renal failure, glucocorticoid use, and cytotoxic drugs such as cyclophosphamide. The recurrent PRES in this patient was much more severe, culminating in intracranial hemorrhage. Timely diagnosis and appropriate treatment were a challenge for us. While supportive and symptomatic treatment, including blood pressure control and antiepileptic medication, was essential, modifying the immunosuppressive regimen was difficult. Given the declining lupus activity score (excluding the nervous system) and the understanding that PRES can develop even weeks and months after initiation of drug therapy (e.g., application of immunosuppressive drugs after transplantation), glucocorticoid pulse therapy was withheld. Instead, a tapered steroid regimen and cyclophosphamide were continued, resulting in a stable condition subsequently.

This case report has several limitations. First, as a single case, the findings may not be generalizable to all patients with SLE who develop PRES. Second, although the clinical course and radiological findings supported the diagnosis of PRES, we could not completely exclude the possibility of overlapping NPSLE, due to the lack of cerebrospinal fluid analysis or brain biopsy. Third, the pathophysiological mechanisms underlying the recurrence of PRES in this patient remain unclear, and further mechanistic studies or longitudinal case series are needed to clarify the causal relationships between SLE activity, treatment, and PRES relapse. Despite these limitations, this case underscores the need for heightened clinical awareness and timely intervention in similar presentations.

## 4. Conclusion

In SLE patients with PRES, symptoms often overlap with those of NPSLE. Timely diagnosis and appropriate treatment, particularly adjusting immunosuppressive treatment and addressing the underlying toxic condition, can reduce PRES recurrence and improve overall patient outcomes.

## Acknowledgments

I would like to thank all medical staff of the Department of Nephrology.

## Author contributions

**Conceptualization:** Binbin Chen, Tingting Xie.

**Data curation:** Hong Hong, Cong Wang.

**Formal analysis:** Hong Hong, Jie Wang, Cong Wang.

**Funding acquisition:** Cong Wang.

**Methodology:** Xiuzhen Li.

**Project administration:** Xiuzhen Li.

**Writing – original draft:** Binbin Chen.

**Writing – review & editing:** Binbin Chen.
